# Distinct Cell Clusters Touching Islet Cells Induce Islet Cell Replication in Association with Over-Expression of Regenerating Gene (REG) Protein in Fulminant Type 1 Diabetes

**DOI:** 10.1371/journal.pone.0095110

**Published:** 2014-04-23

**Authors:** Kaoru Aida, Sei Saitoh, Yoriko Nishida, Sadanori Yokota, Shinichi Ohno, Xiayang Mao, Daiichiro Akiyama, Shoichiro Tanaka, Takuya Awata, Akira Shimada, Youichi Oikawa, Hiroki Shimura, Fumihiko Furuya, Soichi Takizawa, Masashi Ichijo, Sayaka Ichijo, Jun Itakura, Hideki Fujii, Akinori Hashiguchi, Shin Takasawa, Toyoshi Endo, Tetsuro Kobayashi

**Affiliations:** 1 Department of Internal Medicine III, Interdisciplinary Graduate School of Medicine and Engineering, University of Yamanashi, Chuo, Yamanashi, Japan; 2 Department of Anatomy and Molecular Histology, Interdisciplinary Graduate School of Medicine and Engineering, University of Yamanashi, Chuo, Yamanashi, Japan; 3 Department of Nursing, Interdisciplinary Graduate School of Medicine and Engineering, University of Yamanashi, Chuo, Yamanashi, Japan; 4 Section of Functional Morphology, Faculty of Pharmaceutical Sciences, Nagasaki International University, Saseho, Nagasaki, Japan; 5 Department of Computer Science, Interdisciplinary Graduate School of Medicine and Engineering, University of Yamanashi, Chuo, Yamanashi, Japan; 6 Division of Endocrinology and Diabetes, Department of Medicine, Saitama Medical School, Moroyama, Saitama, Japan; 7 Department of Internal Medicine, Saiseikai Central Hospital, Tokyo, Japan; 8 Department of Laboratory Medicine, Fukushima Medical University, Fukushima, Fukushima, Japan; 9 Department of Surgery I, Interdisciplinary Graduate School of Medicine and Engineering, University of Yamanashi, Chuo, Yamanashi, Japan; 10 Department of Pathology, Keio University School of Medicine, Tokyo, Japan; 11 Department of Biochemistry, Nara Medical University, Kashihara, Wakayama, Japan; Hirosaki University Graduate School of Medicine, Japan

## Abstract

**Background:**

Pancreatic islet endocrine cell-supporting architectures, including islet encapsulating basement membranes (BMs), extracellular matrix (ECM), and possible cell clusters, are unclear.

**Procedures:**

The architectures around islet cell clusters, including BMs, ECM, and pancreatic acinar-like cell clusters, were studied in the non-diabetic state and in the inflamed milieu of fulminant type 1 diabetes in humans.

**Result:**

Immunohistochemical and electron microscopy analyses demonstrated that human islet cell clusters and acinar-like cell clusters adhere directly to each other with desmosomal structures and coated-pit-like structures between the two cell clusters. The two cell-clusters are encapsulated by a continuous capsule composed of common BMs/ECM. The acinar-like cell clusters have vesicles containing regenerating (REG) Iα protein. The vesicles containing REG Iα protein are directly secreted to islet cells. In the inflamed milieu of fulminant type 1 diabetes, the acinar-like cell clusters over-expressed REG Iα protein. Islet endocrine cells, including beta-cells and non-beta cells, which were packed with the acinar-like cell clusters, show self-replication with a markedly increased number of Ki67-positive cells.

**Conclusion:**

The acinar-like cell clusters touching islet endocrine cells are distinct, because the cell clusters are packed with pancreatic islet clusters and surrounded by common BMs/ECM. Furthermore, the acinar-like cell clusters express REG Iα protein and secrete directly to neighboring islet endocrine cells in the non-diabetic state, and the cell clusters over-express REG Iα in the inflamed milieu of fulminant type 1 diabetes with marked self-replication of islet cells.

## Introduction

During research into the destruction and regeneration of islet cells in fulminant type 1 diabetes (FT1DM) [1]–[3], impressive pancreatic acinar-like cell clusters over-expressing regenerating (Reg) gene protein Iα (REG Iα) [4] have been found just beside islet cell clusters. We first studied the anatomical relationship between the basement membranes (BMs) and extra cellular matrix (ECM) surrounding islet cell clusters and the acinar-like cell clusters around the islets that express REG Iα proteins in non-diabetic human pancreas. Then, the topographic relationship between islet cell clusters and acinar-like cell clusters, which are present around islet cell clusters and express REG Iα proteins, was studied. Finally, *in situ* changes in REG Iα-expressing acinar-like cell clusters, islet vasculature, and BMs/ECM around the islets in the inflamed milieu of FT1DM were examined.

The capsule comprising BMs and ECM surrounding mature islet cell clusters is vital for their normal growth and renewal, and for protection against inflammation, especially from type 1 diabetes [5], [6]. In addition, BMs and ECM surrounding islet cell clusters are increasingly important because they have a major effect on engraftment in islet cell transplantation [7]. Furthermore, beta cell tropic factors, including regenerating (Reg) gene proteins [4] and other growth factors, are expressed in exocrine pancreas cells near the islets [8]. Recent studies have demonstrated that progenitor cells of islet beta cells potentially reside in the exocrine (acino-ductal) pancreas [8]. Adult human pancreatic islets and pancreatic exocrine cells are assumed to be covered by their own capsules and are separated from each other, making it difficult for them to communicate directly [9]–[11]. However, the precise topographic and physiological relationships between the islets and the nearby exocrine cell clusters remain completely unclear in humans.

FT1DM is characterized by abrupt-onset diabetes usually related to viral infection, followed by accelerated innate and adaptive immune reactions [1]–[3], [12], [13].

We demonstrated that, in the non-diabetic state, islet cell clusters and pancreatic acinar-like cell clusters adhere directly to each other and are encapsulated by continuous BMs and ECM. The acinar-like cells exocytosed REG Iα molecules to islet cells directly. In the inflamed milieu of FT1DM, an increased number of Ki67-positive regenerating islet cells in contact with REG Iα-over-expressing acinar cell-like clusters was observed. Acinar-like cell clusters packed with islet cell clusters may have specific roles in islet cell replication.

## Research Design and Methods

### Patients

The clinical profiles of three autopsied patients with FT1DM (cases 1–3) have been reported previously [2], [3]. Briefly, case 1 was a 14-year-old boy who died of diabetic ketoacidosis (DKA) following flu-like symptoms 5 days earlier. Case 2 was a 25-year-old man who died of DKA following sudden symptoms of nausea and epigastralgia 2 days earlier. Case 3 was a 29-year-old man who died of DKA following slight fever, nausea, and vomiting 2 days earlier.

### Non-diabetic control subjects

Pancreatic tissues from 10 non-diabetic men (62±10 years, mean ± SD) with gastric carcinoma who had undergone partial pancreatectomy and from five autopsied non-diabetic men (65±11 years) were used as non-diabetic control subjects for immunohistochemical analyses. Male age-matched non-diabetic control pancreata (29±13 years, n = 16) were recruited to compare the frequencies of Ki67-positive cell in the pancreas.

Pancreatic tissues from patients with chronic pancreatitis (M/F: 3/2, 55±21 years, n = 5) and type 2 diabetes (M/F: 4/2: 74±10 years, n = 6) were recruited to examine the cyto-structure with REG Iα expression.

For the serum REG Iα assay, sera from 11 patients with FT1DM (M/F: 7/4, age: 52±6 years) and 20 non-diabetic controls (M/F: 14/6, age: 42±6 years) were collected. All FT1DM samples were taken less than 2 weeks after the onset of diabetes.

### Immunostaining and topographic analysis of the pancreas

The methods for immunohistochemical analyses have been reported previously [2], [3]. The primary antibodies used in this study are listed in Table S1. The definition of insulitis and the frequencies of insulitis and mononuclear cell (MNC) phenotypes in islets of cases 1–3 have been documented previously [2], [3]. A confocal laser-scanning microscope, Fluoview FV1000 (Olympus, Tokyo, Japan), was also used.

Analyses of capsules containing BMs and ECM that surround islets and exocrine pancreatic tissues were performed using specific antisera against fibronectin and type IV collagens, and laminin (Table S1). Histological analyses were performed on at least 80 randomly selected islets/case from the tissue sections.

Morphometric analyses were performed using NIH Image software (http://rsb.info.nih.gov/nih-image/). Photographs of histological specimens for each case were taken at magnifications of ×200 and ×400 for analysis. 3D analysis of the BMs surrounding the islets and acinar-like cell clusters was principally performed as previously reported [14]. The human pancreatic tissues were fixed in 4% paraformaldehyde and cut as serial sections at depths of ≤4 µm. A total of 40 pancreatic sections were reconstructed for Z-axis optical sections with 10× objective lenses to acquire the images. A 3D animation was generated with 3DS Max (Autodesk, Inc., San Francisco, CA, USA) based on the cross-sectional images for providing an intuitive illustration of the 3D structure.

Ki67-positive cells were counted in the islets of FT1DM and control cases. From 16 non-diabetic pancreata, a total of 215 islets (13±2 islets/person, mean ± SD, range: 8–15), and from 3 fulminant type 1 diabetic pancreata, a total of 72 islets (24±5 islets/person, range: 18–28) were analyzed. From 16 non-diabetic pancreata, a total of 17,060 beta cells (1066±260 beta cells/islet, mean ± SD, range: 709–1629), and from 3 fulminant type 1 diabetic pancreata, a total of 417 beta cells (139±222 beta cells/islet, mean ± SD, range: 7–395) were analyzed.

### Electron and immune-electron microscopy

#### Tissue preparation for transmission electron microscopy

Small pieces of human pancreatic tissues from 10 patients were obtained during pancreatic resection in surgical operating rooms. The tissue specimens were pre-fixed with 2.5% glutaraldehyde in 0.1 M phosphate buffer, pH 7.4 for 1 h and post-fixed with 1% osmium tetroxide in 0.1 M PB for 1 h. They were dehydrated in a graded series of ethanol and then embedded in Epon 812 epoxy resin.

To examine the specimens, thick sections were first cut at a thickness of 0.5 µm and routinely stained with toluidine blue. Then, ultrathin sections were serially cut at a thickness of 70–80 nm with a diamond knife on an ultramicrotome, mounted on copper grids, and doubly stained with uranyl acetate and lead citrate. They were finally observed under a transmission electron microscope at an accelerating voltage of 80–100 kV (H-7500; Hitachi, Tokyo, Japan, JEM-1400; JOEL, Tokyo, Japan).

#### Immuno-electron microscopy

Surgically removed specimens of human pancreas were fixed immediately after removal of tissues in fixative composed of 4% paraformaldehyde, 0.2% glutaraldehyde, 0.02% CaCl_2_, and 0.05 M HEPES-KOH buffer (pH 7.4) at 4°C for 1–2 h. After washing in PBS, small tissue blocks of pancreas were dehydrated with a graded series of ethanol concentrations for embedding in LR White (London Resin, Reading, UK). Semi-thin sections (1-µm-thick) of LR White-embedded pancreatic tissue were cut with a diamond knife equipped with a Reichert Ultracut R, mounted on a glass slide, and stained with 0.1% toluidine blue. Langerhans islets in the pancreas were identified and trimmed. Thin sections of the islets were cut and mounted on naked nickel grids and stored in a desiccator. Single staining of antigens was carried out as follows. Both sides of sections were treated with 2% fish gelatin in PBS for 30 min and incubated in droplets of primary antibodies including rabbit anti-REG I, guinea pig anti-insulin, and mouse anti-amylase, separately overnight at 4°C, followed by a 30-min incubation with protein A/G/L-gold (15 or 25 nm). For double staining, one side of the sections was stained by a combination of anti-REG Iα and a 25-nm gold probe, and the other side was stained by a combination of anti-insulin and a 5- or 8.5-nm gold probe. For a control, the primary antibody was omitted, followed by gold probes. All sections were contrasted and then examined with a Hitachi H7650 electron microscope (Hitachi, Tokyo, Japan) at an acceleration voltage of 80 kV.

### REG Iα assay

The serum levels of REG Iα were assayed using ELISA kits (BioVender-Laboratorni Medica, Co., Brno, Czech Republic, normal range: 75–1222 ng/ml). Intra-assay and inter-assay coefficients of variation (CVs) were 2% and 5%, respectively.

### Ethics

The Ethics Committee of the University of Yamanashi approved all of the procedures performed in this study. All patients gave their written informed consent for examination of the resected pancreas and serum samples. Witten informed consent was obtained from the next of kin or parents/guardians on behalf of the children or autopsied cases. The informed consent was written on the form and kept in the medical records. The Ethics Committee of the University of Yamanashi approved the consent procedure.

### Statistical analysis

Differences between groups were compared using Student's *t*-test and ANOVA. Fisher's exact test was used to compare islet frequencies. Values are expressed as means ± SD unless otherwise noted.

## Results

### Pancreatic acinar-like cell clusters are packed with Langerhans islet cell cluster and surrounded by continuous BMs and ECM in humans

**Figure 1:**
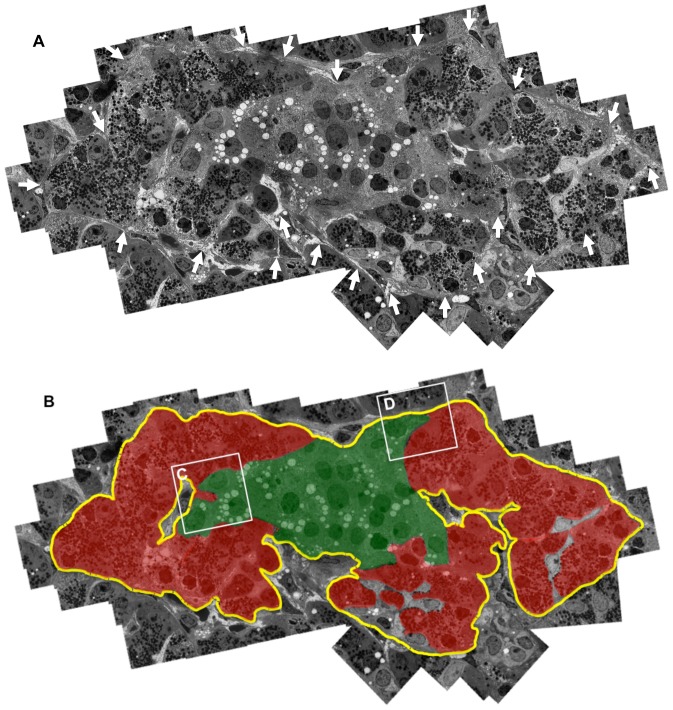
Demonstration of pancreatic acinar-like cell clusters touching islet-cell clusters that are covered by a common capsule. **A:** Continuous basement membranes (BMs) and extracellular matrix (ECM) (arrows) cover the two cell clusters. A combined figure of 65 electron microscopic photos is shown. **B:** Schematic demonstration of Figure 1A. The yellow line indicates continuous BMs and ECM surrounding islet cell (green) and acinar-like cell (red) clusters. LB: lipofuscin body.

**Figure 2:**
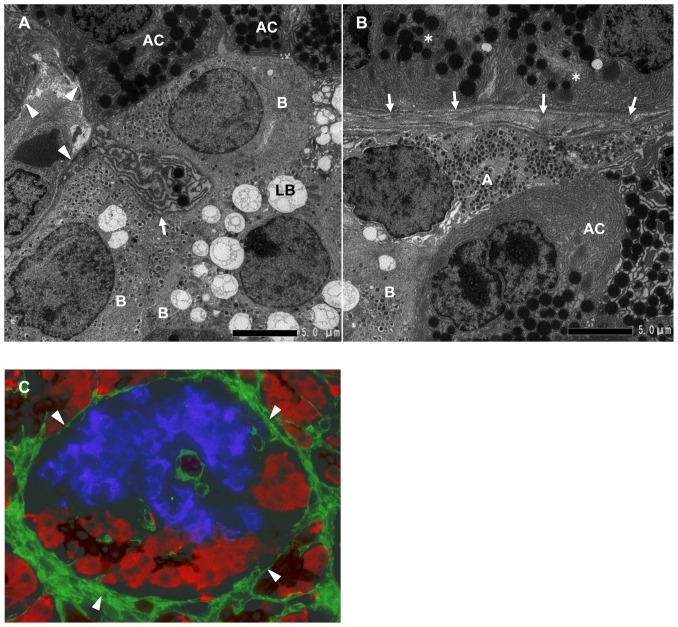
The interface between acinar-like cell clusters and islet cell clusters. **A**: Magnified view of the interface between acinar-like cell clusters and islet cell clusters shown in Figure 1B (inset C). Acinar-like cells (AC) contact beta cells (B). Note that the acinar-like cell has a process (arrow) containing vesicles that protrude to the beta cell cytoplasm. BMs and ECM (arrowheads) surround beta cells (B) and acinar-like cells (AC). **B**: Magnified view of the interface between acinar-like cell clusters and islet cell clusters shown in Figure 1B (inset D). Alpha cell (A) and beta cell (B) touching an acinar-like cell (AC) and the covering BMs and ECM (arrows) and pancreatic acinar cells (*) separated by BMs and ECM (arrows). **C**: Immunohistological demonstration of BMs and ECM stained for fibronectin (arrowheads, green), surrounding the islet beta cells stained for insulin (blue), and acinar-like cells (red) stained for amylase and the ductal marker cytokeratin 19 (brown).

Electron microscopy clearly demonstrated continuous BMs and ECM composed of lamina lucida, lamina densa, and lamina reticularis, and fibrous ECM surrounding some acinar-like cell clusters and islet cell clusters (Figure 1A, B, Figure 2A, B).

Immunohistochemical staining also showed that islet cell clusters and pancreatic acinar-like cell clusters were surrounded by continuous BMs and ECM composed of fibronectin, laminin, and type IV collagen, and acinar-like cell clusters marked by amylase were localized just beside islet cell clusters (Figure 2C, Figure S1A, B). Silver staining of the human pancreas also showed that BMs and ECM were not observed at the interface between islet cell clusters and acinar-like cell clusters (data not shown). In islet cell clusters, BMs of the vasculature were seen to have a double membrane pattern characteristic of humans (Figure S1A, B) [15]. Some acinar-like cell clusters were associated with cytokeratin (CK)-19-positive ductal-like cells (Figure 2C).

**Figure 3:**
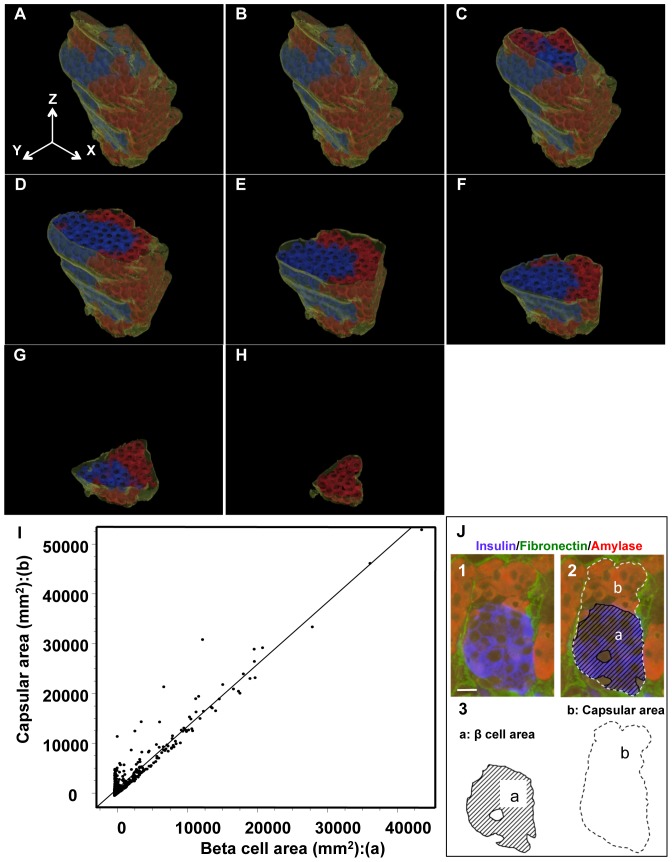
Reconstructed feature of islet cell cluster, acinar-like cell cluster, and BMs and ECM. **A–H**: Overview of the architecture reconstructed by serial pancreatic beta cell clusters (insulin: blue) and acinar-like cell clusters (amylase: red) surrounded by continuous BMs and ECM (fibronectin: green). **I, J**: Relationship between the beta cell area (X-axis) and the capsular area (Y-axis), which contains the beta cell area and the acinar-like cell area. A clear linear correlation between beta cell area and capsular area was observed (p<0.001, n = 517). See the schematic definition in (J). **J 1–3**: Schematic definition of capsular area and acinar-like cell area. The capsular area (b: dashed line) is composed of the acinar-like cell area (red in the dashed line) and the islet cell area (a: blue) (mostly beta cell area). A good correlation between the capsular area and islet cell area (Figure 3-I) indicates that a constant proportion of islet cell clusters are accompanied by acinar-like cell clusters irrespective of the size of beta cell clusters.

3D-reconstruction with serial thin pancreatic sections of human pancreas showed that acinar-like cell clusters were packed with islets and surrounded by continuous BMs and ECM (Figure 3A–H, Video S1, Figure S2).

The frequency of the presence of acinar-like cell clusters together with islets, calculated from the sections of 5 non-diabetic subjects, was 52±17% (range 30∼76%, n = 508). A clear linear correlation between the areas of beta cell clusters and the areas of capsules composed of BMs and ECM, which included acinar-like cell clusters and beta cell clusters, was observed (Figure 3I, J).

### Cell communications between pancreatic acinar-like cell clusters and islet cell clusters, which are packed within a continuous capsule composed of BMs and ECM

**Figure 4:**
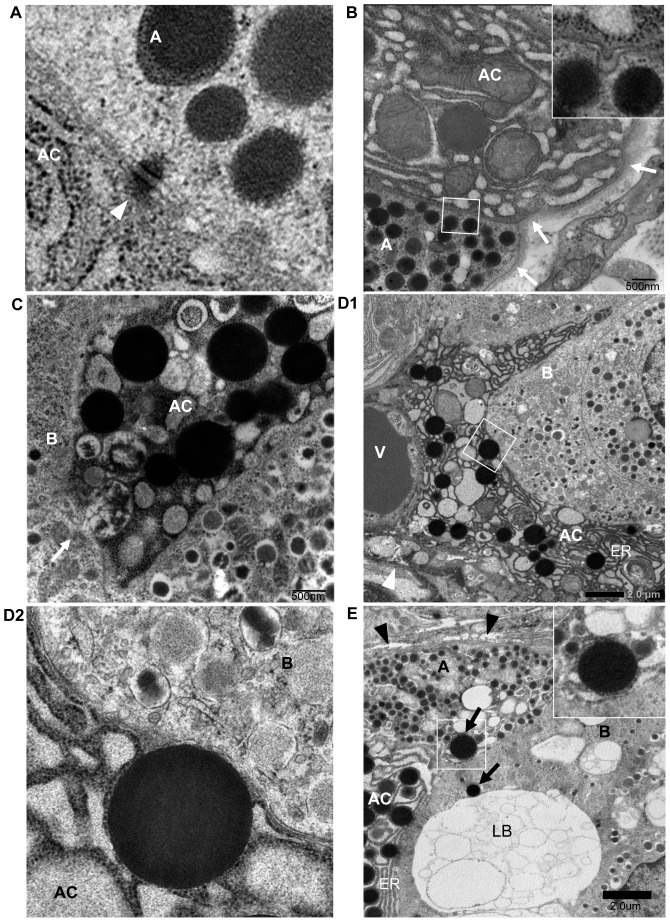
Cell-cell interaction between acinar-like cells and islet endocrine cells encapsulated by common BMs. **A:** A desmosomal junction (arrowhead) is observed between acinar-like cells (AC) and alpha cells (A), which are encapsulated by common BMs and ECM. **B**: Coated-pit-like structure is observed between an acinar-like cell (AC) and an alpha cell (A) touching directly and covered by common BMs (arrows). Inset shows a magnified view of the coated-pit-like structure. **C**: Excretion of vesicles from acinar-like cells (AC) to a beta cell (B). Note the vesicular membrane of the AC is dissolved (arrow), and the vesicular content is released to the beta cell (B) touching it. **D1**: Exocytotic features of vesicles in acinar-like cells (AC) to beta cells (B), which are in contact with each other. Arrowhead indicates BMs/ECM encapsulating acinar-like cells and beta cells, V: vasculature. **D2:** Higher magnified view of D1. The vesicle is internalized to the beta cell. AC: acinar-like cell, B: beta cell. **E:** Vesicles of acinar-like cells (AC) are internalized to touching alpha cells (A) and beta cell (B) shown by arrows. Arrowheads indicate BMs/ECM surrounding beta cell (B), alpha cell (A), and acinar-like cell (AC). Inset shows magnified view of (E). LB: lipofuscin body, ER: endoplasmic reticulum.

Electron microscopy demonstrated that acinar-like cells in the same capsule with islet cell clusters were in contact with islet cells, including beta cells and alpha cells (Figures 1A, B, 4A, B). Between acinar-like cells and islet endocrine cells, desmosomal structures were found (Figure 4A). Between these cell interfaces, coated-pit-like structures were observed (Figure 4B).

Secretory vesicles in acinar-like cells were arranged along the interfaces between islet cells. Acinar-like cells had characteristic spike-like processes that protruded to islet cells (Figures 2A, 4C). Some secretory vesicles in acinar-like cells were exocytosed to closely touch beta cell walls in the non-diabetic state, and some vesicles in acinar-like cells exocytosed vesicles directly to the contacting beta cells (Figure 4C, D). Some vesicles of acinar-like cells were present in contacting beta cells and alpha cells (Figure 4E).

Considering the functional roles of acinar-like cell clusters with respect to islet cell clusters, the Acinar-like cell cluster Touching Langerhans islets with Thin Interstitial Surrounding is hereby abbreviated as ATLANTIS.

### REG Iα molecules are expressed in the cytoplasm of ATLANTIS in the physiological state

**Figure 5:**
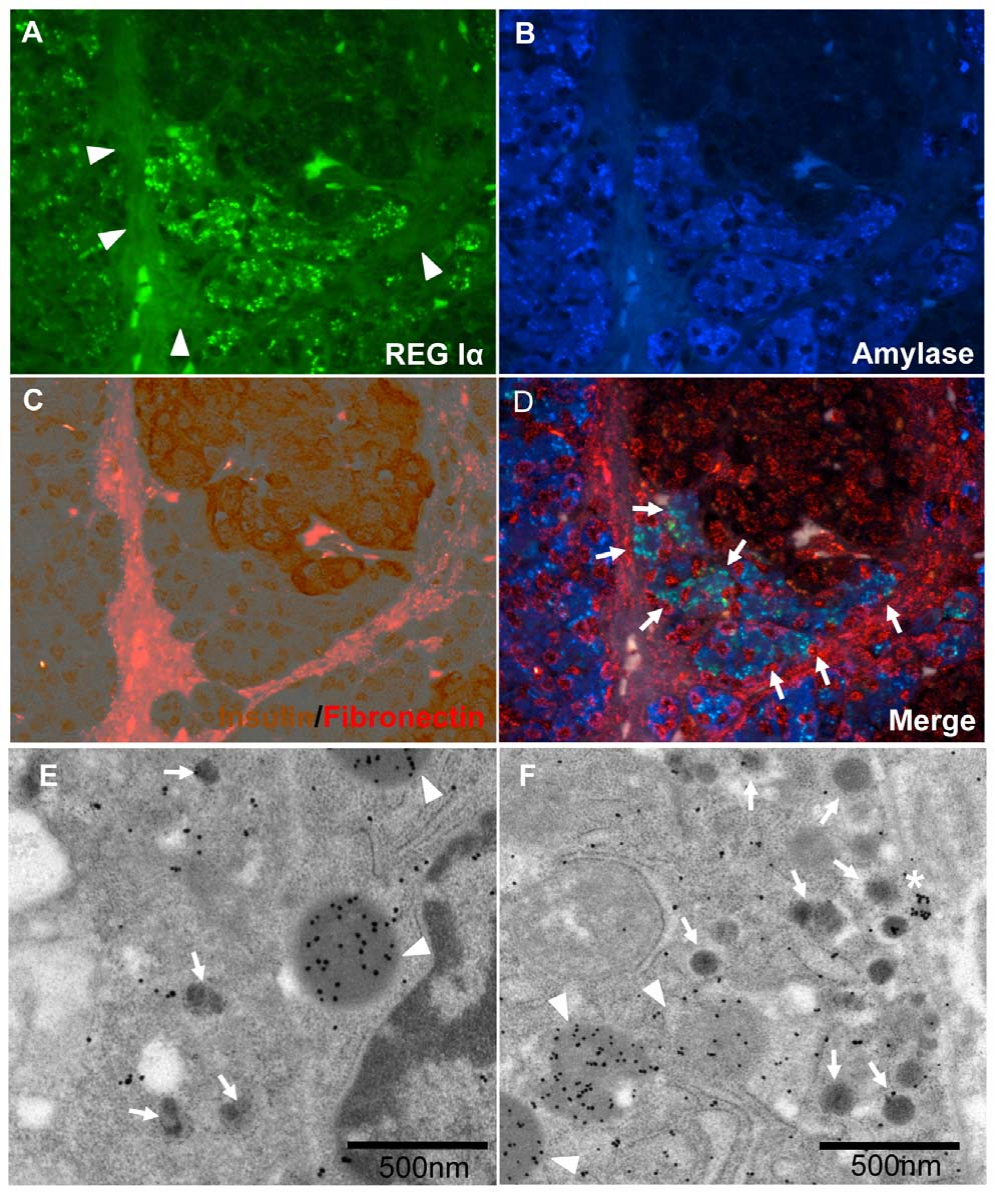
REG Iα-positive cell cluster contacting with beta cell cluster and surrounded by common BMs and ECM. **A–D**: Immunostaining for REG Iα (green) (**A**), acinar-like cell marker, amylase (blue). (**B**), and double immunostaining with insulin (brown) and fibronectin (red) (**C**). Auto-fluorescence of collagen fiber surrounding islet is observed (**A**, arrowheads). The merged image (**D**) shows that amylase-positive acinar-like cells that are in contact with beta cells express REG Iα protein (light blue: arrowheads), and the acinar-like cell cluster is surrounded by BMs/ECM (red, fibronectin). **E**: Electron-immunostaining with immuno-gold for REG Iα (20 nm: arrowheads) in acinar-like cell touching a beta cell containing insulin (5 nm: arrows). REG Iα is mainly localized in the center of the vesicle that is near the beta cell wall. **F**: Immuno-electron microscopy with immunogold for REG Iα (25 nm: arrowheads) and insulin (5 nm: arrows). Densely stained REG Iα vesicle (*) is just beside the cell wall touching a beta cell. Dissolved vesicles positive for REG Iα (arrowheads) are observed in insulin-containing beta cells.

In the non-diabetic state, REG Iα was mainly expressed in the cytoplasm of ATLANTIS (Figure 5A–D). Weaker immunostainings for REG Iα were observed in the exocrine pancreas around islets (Figure 5A). Immuno-electron microscopic examination showed that REG Iα was detected mainly in the center core of vesicles of ATLANTIS (Figure 5E). Furthermore, REG Iα vesicles were present in insulin-positive beta cells (Figure 5F).

### BMs and ECM encapsulating islet cells and ATLANTIS were disrupted in FT1DM

**Figure 6:**
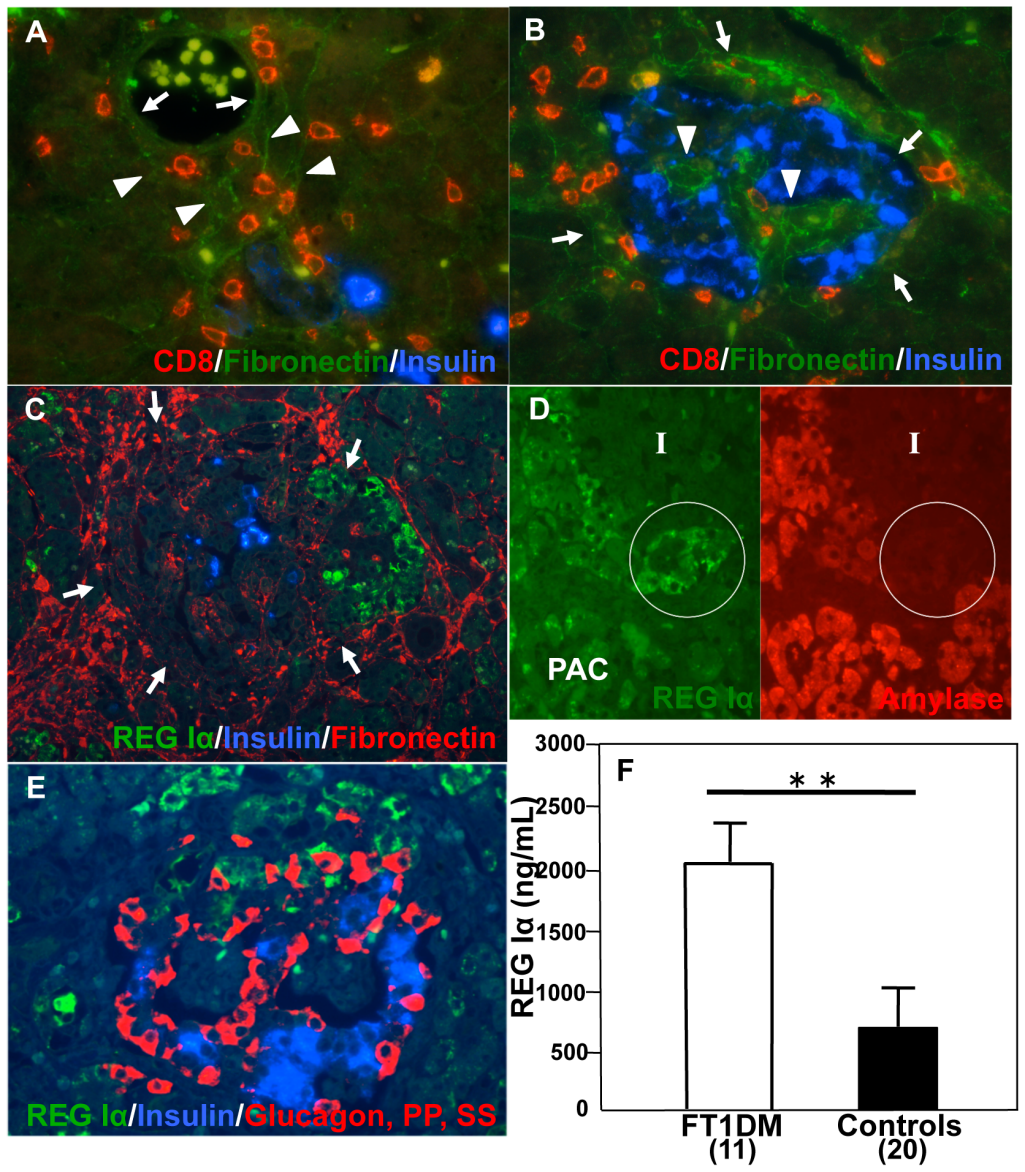
Pathological features of the pancreas affected by fulminant type 1 diabetes (FT1DM). **A:** CD8 + T cells (red) infiltrate from outside the islet, disrupting vascular BMs (green, arrows) through the interstitial space between the vasculature and islets. Arrowheads indicate BMs (green) of exocrine pancreatic cells. **B:** BMs and ECM surrounding islets (green) are markedly disrupted and punctuated (arrows) in FT1DM. The vasculatures of the islets show marked dilation, and the vascular BMs have lost the human-specific double membrane profile [15] (arrowheads). **C:** Acinar-like cell cluster touching Langerhans islets with thin interstitial surrounding (ATLANTIS) shows marked expression of REG Iα (green) in FT1DM. BMs (red, arrows) encapsulating the islet beta cells (blue) and ATLANTIS (green) are disrupted and discontinuous in some parts. **D:** Double immunostaining for amylase (red) and REG Iα (green) shows that amylase expression in the ATLANTIS (in circle) in inflamed FT1DM becomes faint in inverse relation to REG Iα over-expression. I: Islet, PAC: pancreatic acinar cells. **E:** Triple immunostaining for REG Iα (green), glucagon + somatostatin (SS) + pancreatic polypeptide (PP) (red), and insulin (blue) demonstrates that REG Iα-positive cells are not beta, glucagon, SS, or PP cells. **F:** Serum levels of REG Iα are increased in the patients with FT1DM of duration less than 2 weeks. **p<0.01 vs. controls.

CD8^+^ T cells and CD68^+^ macrophages were observed in the interstitial space outside the vasculature (Figure 6A). The interstitial spaces were bounded by BMs of the exocrine pancreas and of vasculature (Figure 6A).

The BMs and ECM of the capsules surrounding the islet cell clusters and ATLANTIS were markedly disrupted and punctuated in FT1DM (Figure 6B).

### Over-expression of REG Iα in ATLANTIS with increased serum levels of REG Iα in FT1DM

In FT1DM pancreas specimens with marked insulitis, cell clusters of ATLANTIS, which were in contact with islet cells, strongly expressed REG Iα molecules (Figure 6C). In contrast, the expression of amylase, which was stained in ATLANTIS in the non-diabetic state, was markedly decreased in FT1DM (Figure 6D). REG Iα expression was not observed in islet endocrine cells of FT1DM patients (Figure 6E). Over-expression of REG Iα was observed in chronic pancreatitis in both ATLANTIS cells and pancreatic acinar cells (Figure S3). Over-expression of REG Iα was not observed in both ATLANTIS cells and pancreatic acinar cells in type 2 diabetes. Serum levels of REG Iα were markedly elevated in patients with FT1DM at the onset of diabetes (Figure 6F). Over-expression of REG IIIα and REG IV was not observed in the pancreas of FT1DM (Figure S4). Expression of EXTL3, putative REG Iα receptor, was observed in beta cells of FT1DM, chronic pancreatitis, type 2 diabetes and non-diabetic control (Figure S5).

### Increased Ki67^+^ islet cells in the pancreas in FT1DM

**Figure 7:**
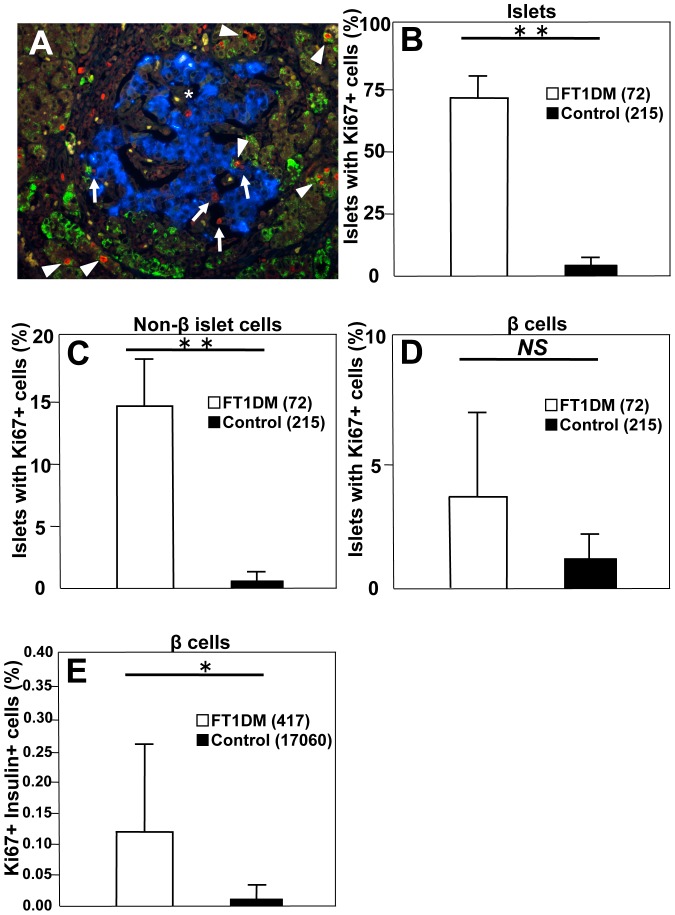
Replicating islet cells in fulminant type 1 diabetes (FT1DM). **A**: Ki67-positive cells are increased in the pancreas of FT1DM. The cell composition of increased Ki67^+^ cells (red nuclei) is mainly REG Iα positive cells (green: arrowheads), islet non-beta cells (arrows), and islet beta cells (*, blue). **B**: The percentage of islets positive for Ki67 is increased in the pancreas of FT1DM. The number in parentheses indicates the total number of islets studied in FT1DM and control. See the detailed characterization of the subjects in Research Design and Methods. **P<0.0001 vs. controls, mean ± SEM. **C**: The number of islets positive for Ki67 in non-beta islet cells is increased in FT1DM. Non-beta islet cells were stained by mixed antisera for glucagon, somatostatin, and pancreatic polypeptide, and the values are expressed as percentage of islets positive for Ki67. The number in parentheses indicates the total number of islets studied in FT1DM and controls. **P = 0.02 vs. controls, mean ± SEM. **D**: The number of islets positive for Ki67 in beta cells tends to increase, but it is not significant due to the markedly decreased number of beta cells in individual islets in FT1DM. The number in parentheses indicates the total number of islets studied in FT1DM and controls. **E**: The number of Ki67-positive beta cells is increased in FT1DM. The numbers in parentheses indicate the total number of beta cells counted in the islets of FT1DM and controls. See a detailed characterization in Research Design and Methods. *P = 0.043 vs. controls, mean ± SEM.

The number of Ki67^+^ cells was increased in the islets of FT1DM (Figure 7A and B). The numbers of islets positive for Ki67 in non-beta cells were markedly increased, including in glucagon-, somatostatin-, and pancreatic polypeptide-positive cells (Figure 7C). The numbers of Ki67^+^ and REG Iα^+^ cells were increased in the ATLANTIS cells (Figure 7A). The number of islets positive for Ki67 in beta cells tended to be higher than in controls, but the difference was insignificant due to the extremely reduced number of beta cells in the islets of FT1DM (Figure 7D). The percentage of beta cells positive for Ki67 was significantly higher in FT1DM than that in age-matched controls (Figure 7E).

## Discussion

The ATLANTIS was located beside the islet cell cluster and secreted REG Iα protein to islet cells. The two cell clusters, islet cells and ATLANTIS, were encapsulated by continuous BMs and ECM. In the inflamed milieu of type 1 diabetes, it was observed that ATLANTIS over-expressed REG Iα protein and islet cells were replicated.

It has been assumed that islet endocrine cell clusters are independent of surrounding pancreatic exocrine tissues and separated from them by capsules composed of BMs and ECM in some species, including humans [10], [11], [15]. The precise structure of capsules around the islets varies across species [16]. In canine pancreas, peri-insular capsules completely cover the islet cells, while the capsules of the islets in human and rodents are extremely thin or discontinuous in parts [11], [16]–[19]. To the best of our knowledge, anatomical packing of islet cell clusters and acinar-like cell clusters within the same space and demonstration of a direct effect of REG Iα on islet cells have not been previously documented. It is conceivable that islet cell clusters and ATLANTIS are encapsulated by continuous BMs and ECM, because the two clusters grew from ductal organs covered by their original continuous BMs in the fetal period. Fibronectin, laminin, and type IV collagens were used as markers of capsules composed of BMs and ECM, which surround islet and acinar-like cell clusters [15], [20]–[22]. Fibronectin, laminin, and type IV collagens are involved in the adhesion and spread of fetal and adult islet cells, and the fibronectin- and laminine-containing pancreatic ECM forms the template for developing islets [20]–[22].

The physiological role of ATLANTIS in the non-diabetic state appears related to maintaining beta cells and other subsets of islet endocrine cells through secreting REG Iα proteins in a paracrine/autocrine manner, filling the microenvironment containing islet cells and ATLANTIS covered by distinct BMs and ECM (Figure 4C and D). REG Iα-knockout mice show decreased islet cell replication [23]. In the inflamed milieu of FT1DM, in which islet beta cells and non-beta cells were damaged, REG Iα proteins were over-expressed in the ATLANTIS. This suggests that ATLANTIS and BMs and ECM encapsulating islets are a growth niche for both islet cell replication and putative islet stem cell differentiation. In fact, FT1DM islet endocrine cells in the same capsule showed marked proliferation via increased numbers of Ki67^+^ islet cells, although islet cell replication should be cautiously interpreted due to possible bias from the small number of samples. As shown in our previous reports [2], [3], inflammatory cytokines and subsequent products of cytokine cascades are rich in the islets in FT1DM. The *REG Iα* gene has an IL-6 responsive element at the upstream region [24], and pro-inflammatory cytokines including interferon (IFN)γ, TNFα, IL-12, and IL-18 have been shown to up-regulate the expression of REG Iα in pancreatic acinar AR42J cells [25], [26]. Thus, it is conceivable that REG Iα responded to the inflamed milieu and produced exaggerated amounts of REG Iα protein to promote the regeneration of damaged islet beta cells. It has already been reported that islet cells, at least beta cells, have REG protein receptors and are affected in an autocrine/paracrine manner [27]. Expressions of REG family proteins, including REG IIIα and REG IV, have been reported in the type 1 diabetic condition (insulitis) in rodent models [28]–[30]. However, in the present study, over-expression of REG IIIα and REG IV was not observed in human type 1 diabetes (Figure S4).

It has been reported that serum levels of REG Iα are increased in chronic pancreatitis and in type 2 diabetes [31], which is similar to FT1DM. We found over-expression of REG Iα in ATLANTIS cells and pancreatic acinar cells in chronic pancreatitis but not in type 2 diabetes immunohistochemically (Figure S4). These findings may highlight a unique feature of over-expression of REG Iα proteins in ATLANTIS cells in FT1DM.

Exocytosis of components in the solubilized or vesicular form of REG Iα molecules was seen from ATLANTIS to islet endocrine cells (Figures 4C, D and E, 5E and F). REG Iα molecules in solubilized form will interact with REG Iα receptors [27] at the surface of beta cells. Direct internalization of REG Iα molecules in the vesicular form to beta cells and alpha cells was also observed. The cell membrane of ATLANTIS fused with the vesicular membrane, and the beta cell membrane is concaved inside, which allowed for entry of REG Iα vesicles into beta cells (Figure 4D). The exocytotic pattern of zymogen granules of pancreatic acinar cells is “sequential fusion” exocytosis, in which multiple granules fuse together and are discharged through the prosome to the ductal lumen [32]–[34], while the excretion pattern of secretory vesicles of ATLANTIS is different, as mentioned above, suggesting the presence of unique cell-cell communication processes between the cells of ATLANTIS and islet cells. The processing, pathway and role of the vesicles containing REG Iα, which were endocytosed into neighboring beta cells, remain speculative. In the endosome, the membrane covers the vesicles (Figure 4D2, Figure 4E) and will keep an acidic and stable condition from pancreatic digestive enzymes that are derived from ATLANTIS cells. In the inflamed condition in FTIDM, the protein production of ATLANTIS cells is converted from the dual production of pancreatic digestive enzymes (e.g. amylase) and REG Iα to dominant REG Iα production (Figure 6D), which is probably related to pro-inflammatory cytokine stimulation as mentioned above. Coated-pit-like structures, which represent receptor-mediated endocytosis, were observed between ATLANTIS cells and islet endocrine cells (Figure 4B). The REG Iα protein in endosomes may bind the corresponding receptor (i.e., EXTL3) [27], [35] and may mediate the signal for regeneration of the endocrine cells. Internal REG Iα receptor(s) (i.e. EXTL3) may also mediate the signal [30], [36]. However, it is unclear how REG Iα molecules delivered to endosome are transported across the endosome membrane into the cytoplasm. In non-diabetic conditions, in which beta cell regeneration by REG Iα is not required, REG Iα-containing vesicles may be processed in the lysosomes (Figure 4E).

The study of ATLANTIS will provide new insights to improve the survival rate of islet function after islet transplantation. A major emphasis in islet transplantation has been the purity of the islet preparation to promote engraftment and reduce immunogenicity [37]. However, a few reports have suggested that islet purification may trigger apoptosis by removing important trophic factors responsible for maintenance and renewal of islet cell viability [38]. The present finding that REG Iα proteins from ATLANTIS packed with islet cell clusters have a vital role in beta cell survival emphasize the need to keep the cell clusters isolated for islet transplantation.

This study demonstrated that BMs and ECM encapsulating islet cell clusters and ATLANTIS were partly disrupted in FT1DM. BMs and ECM are a prerequisite for islet cell attachment and renewal in the non-diabetic state and act as barriers from immune attack in the pathological type 1 diabetes state [6], [39], [40]. Importantly, disrupted microenvironments, especially capsules composed of BMs and ECM that maintain islet cell clusters and ATLANTIS, may impair beta cell replication in type 1 diabetes.

## Supporting Information

Figure S1 **Continuous BMs and ECM encapsulating islets and acinar-like cells.** **A**: Merged image of BMs and ECM stained for laminin (green), insulin (blue), and amylase (red) shows that islet cell clusters and amylase-positive acinar-like cell clusters are surrounded by continuous BMs and ECM (arrowheads). **B**: Merged image of BMs and ECM stained for collagen type IV (green), insulin (blue), and amylase (red) shows that islet cell clusters and amylase-positive acinar-like cell clusters are surrounded by continuous BMs and ECM.
(TIF)

Figure S2 **Serial sections of the pancreas and reconstructed 3D image of an islet cell cluster and acinar-like cell cluster.** Serial sections of the pancreas (1–32) stained for fibronectin as a marker of BMs/ECM (green), acinar-like cells (amylase: red), and beta cells (insulin: blue) and reconstructed 3D image (right column). Vascular BMs in the islets are not shown in 3D image.
(TIF)

Figure S3 **Acinar-like cell clusters touching Langerhans islets with thin interstitial surrounding (ATLANTIS) were found in pancreata of chronic pancreatitis (A) and type 2 diabetes (B).** Over-expression of REG Iα was observed in chronic pancreatitis in both ATLANTIS cells and pancreatic acinar cells (A) but not in both ATLANTIS cells and pancreatic acinar cells of type 2 diabetes (B).
(TIFF)

Figure S4 **No over-expression of REG IIIα and REG IV was observed in the pancreas of FT1DM.** Merged image of REG III (brown) and insulin (red) in FT1DM (A) and non-diabetic control (B). Merged image of REG IV (brown) and insulin (red) in FT1DM (C) and non-diabetic control (D).
(TIFF)

Figure S5 **Expression of EXTL3 (brown), putative REG Iα receptor, was observed in beta cells (red) of fulminant type 1 diabetes (A), chronic pancreatitis (B), type 2 diabetes (C) and non-diabetic control (D).**
(TIFF)

Table S1 **Antibodies used in this study.**
(DOCX)

Video S1 **3D image of islet cluster, acinar-like cell cluster, and BMs and ECM.** 3D demonstration of islet cell clusters (blue), acinar-like cell clusters (red), and BMs and ECM (green). Note that acinar-like cell clusters and islet cell clusters are packed together and surrounded with continuous BMs and ECM.
(AVI)
