# Correction: Distinct Cell Clusters Touching Islet Cells Induce Islet Cell Replication in Association with Over-Expression of Regenerating Gene (REG) Protein in Fulminant Type 1 Diabetes

**DOI:** 10.1371/journal.pone.0105449

**Published:** 2014-08-08

**Authors:** The PLOS ONE Staff

The 11th affiliation for the 20th author is incorrect. Shin Takasawa’s institution is not located in the Wakayama prefecture, but in the Nara prefecture. The correct affiliation should read:

Department of Biochemistry, Nara Medical University, Kashihara, Nara, Japan.

The last sentence of the Figure 1B legend, LB: lipofuscin body, should be the last sentence of the Figure 2A legend. Please see the figures and their corrected legends below.

**Figure 1:**
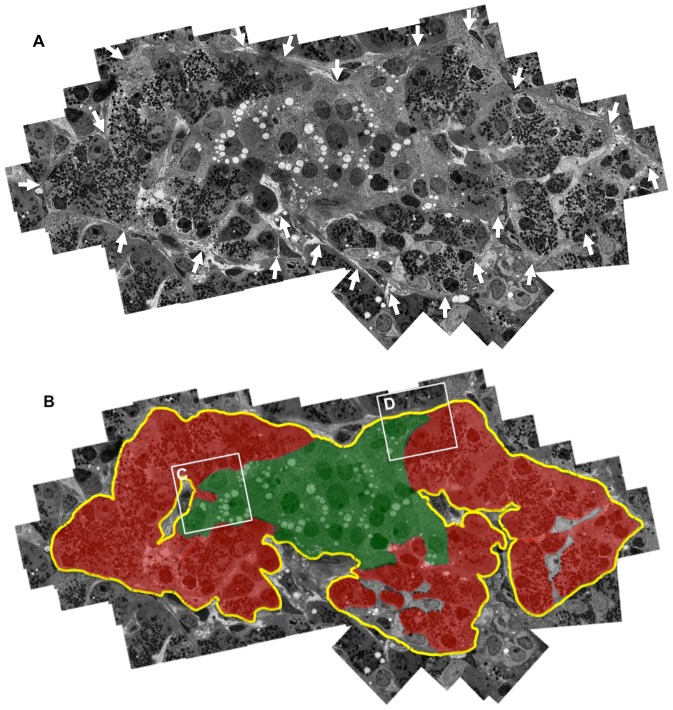
Demonstration of pancreatic acinar-like cell clusters touching islet-cell clusters that are covered by a common capsule. **A:** Continuous basement membranes (BMs) and extracellular matrix (ECM) (arrows) cover the two cell clusters. A combined figure of 65 electron microscopic photos is shown. **B:** Schematic demonstration of Figure 1A. The yellow line indicates continuous BMs and ECM surrounding islet cell (green) and acinar-like cell (red) clusters.

**Figure 2:**
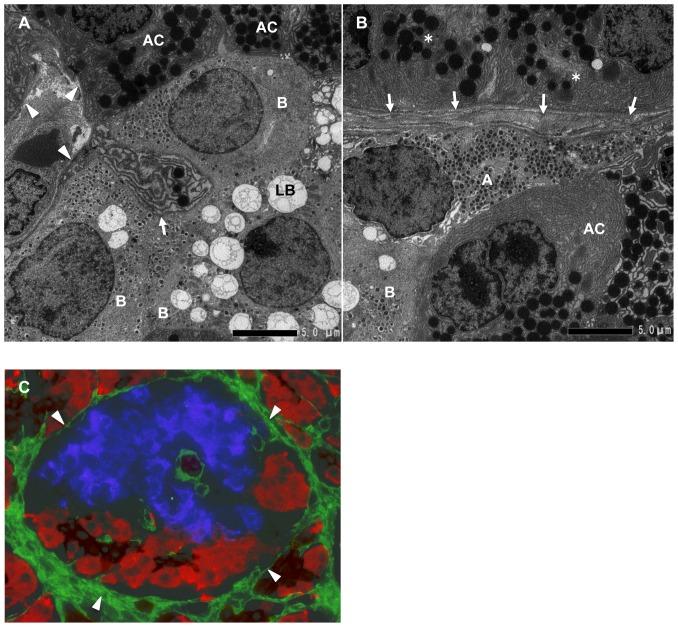
The interface between acinar-like cell clusters and islet cell clusters. **A:** Magnified view of the interface between acinar-like cell clusters and islet cell clusters shown in Figure 1B (inset C). Acinar-like cells (AC) contact beta cells (B). Note that the acinar-like cell has a process (arrow) containing vesicles that protrude to the beta cell cytoplasm. BMs and ECM (arrowheads) surround beta cells (B) and acinar-like cells (AC). LB: lipofuscin body. **B:** Magnified view of the interface between acinar-like cell clusters and islet cell clusters shown in Figure 1B (inset D). Alpha cell (A) and beta cell (B) touching an acinar-like cell (AC) and the covering BMs and ECM (arrows) and pancreatic acinar cells (*) separated by BMs and ECM (arrows). **C:** Immunohistological demonstration of BMs and ECM stained for fibronectin (arrowheads, green), surrounding the islet beta cells stained for insulin (blue), and acinar-like cells (red) stained for amylase and the ductal marker cytokeratin 19 (brown).
