# Supplementary figures and images for: Distinct Cell Clusters Touching Islet Cells Induce Islet Cell Replication in Association with Over-Expression of Regenerating Gene (REG) Protein in Fulminant Type 1 Diabetes
Source: PLoS One. 2014 Apr 23;9(4):e95110. doi: 10.1371/journal.pone.0095110 (PMC3997392; doi:10.1371/journal.pone.0095110)

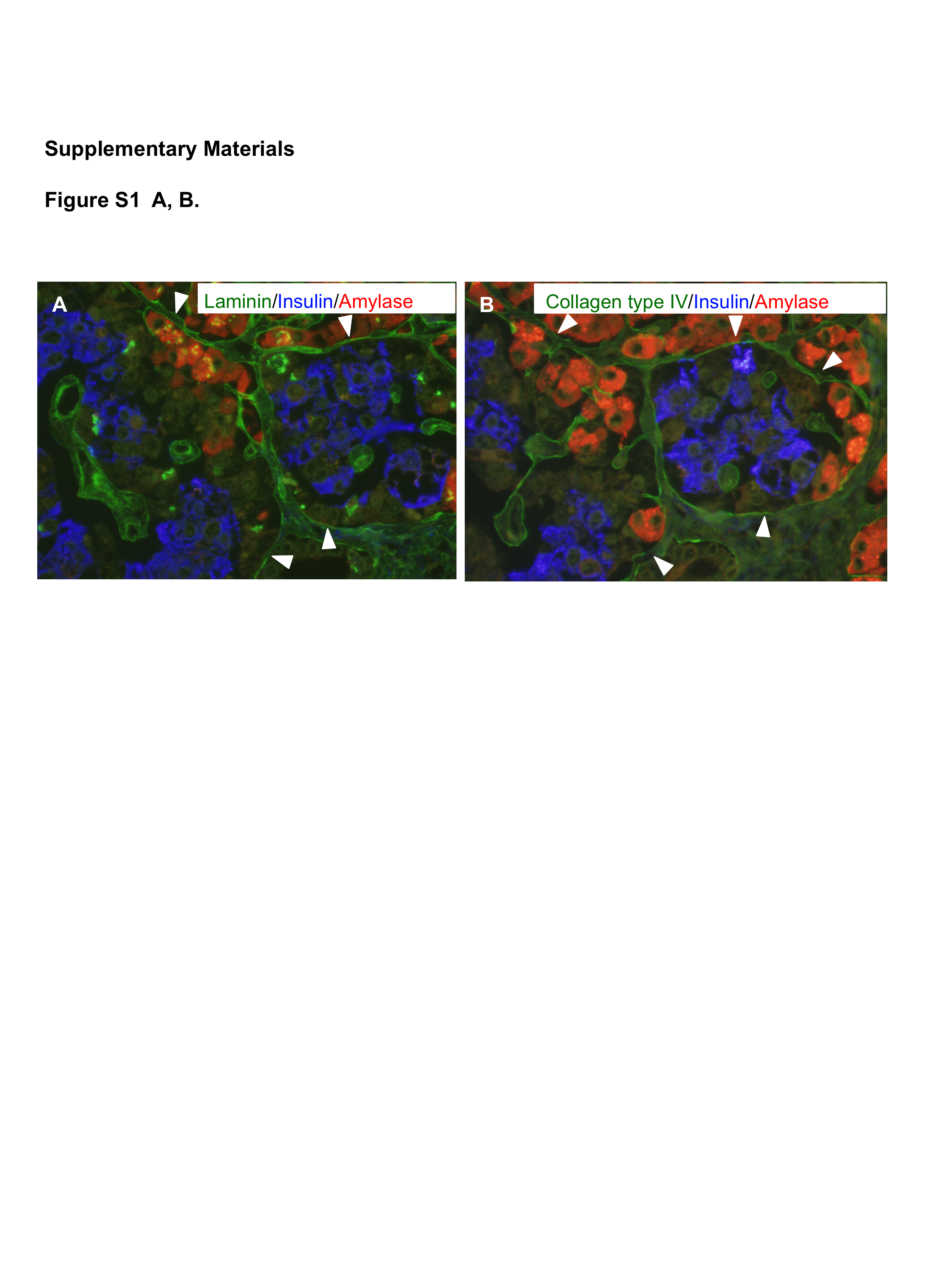

Supplement: Figure S1 — Continuous BMs and ECM encapsulating islets and acinar-like cells. A: Merged image of BMs and ECM stained for laminin (green), insulin (blue), and amylase (red) shows that islet cell clusters and amylase-positive acinar-like cell clusters are surrounded by continuous BMs and ECM (arrowheads). B: Merged image of BMs and ECM stained for collagen type IV (green), insulin (blue), and amylase (red) shows that islet cell clusters and amylase-positive acinar-like cell clusters are surrounded by continuous BMs and ECM. (TIF) [file pone.0095110.s001.tif]

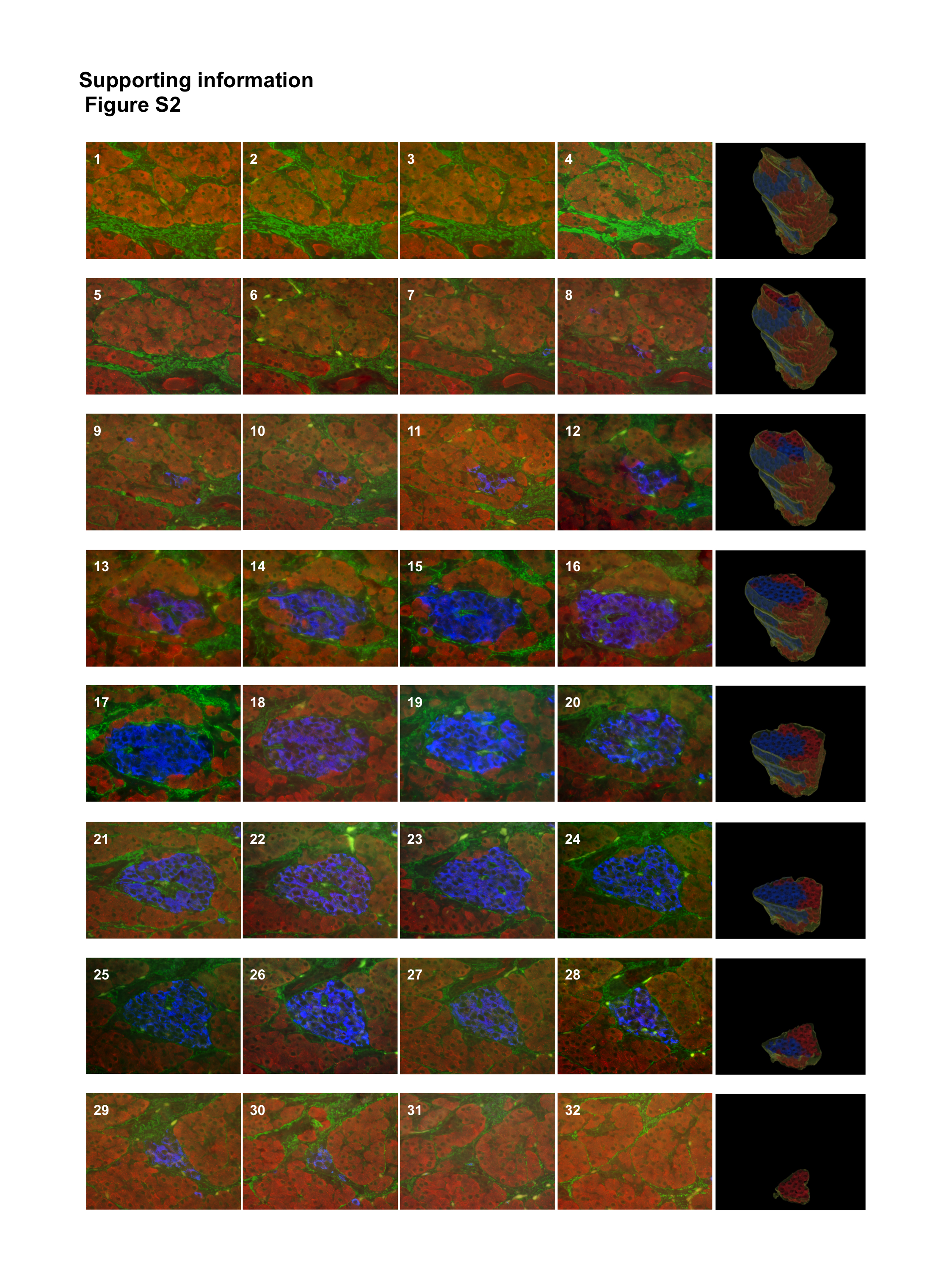

Supplement: Figure S2 — Serial sections of the pancreas and reconstructed 3D image of an islet cell cluster and acinar-like cell cluster. Serial sections of the pancreas (1–32) stained for fibronectin as a marker of BMs/ECM (green), acinar-like cells (amylase: red), and beta cells (insulin: blue) and reconstructed 3D image (right column). Vascular BMs in the islets are not shown in 3D image. (TIF) [file pone.0095110.s002.tif]

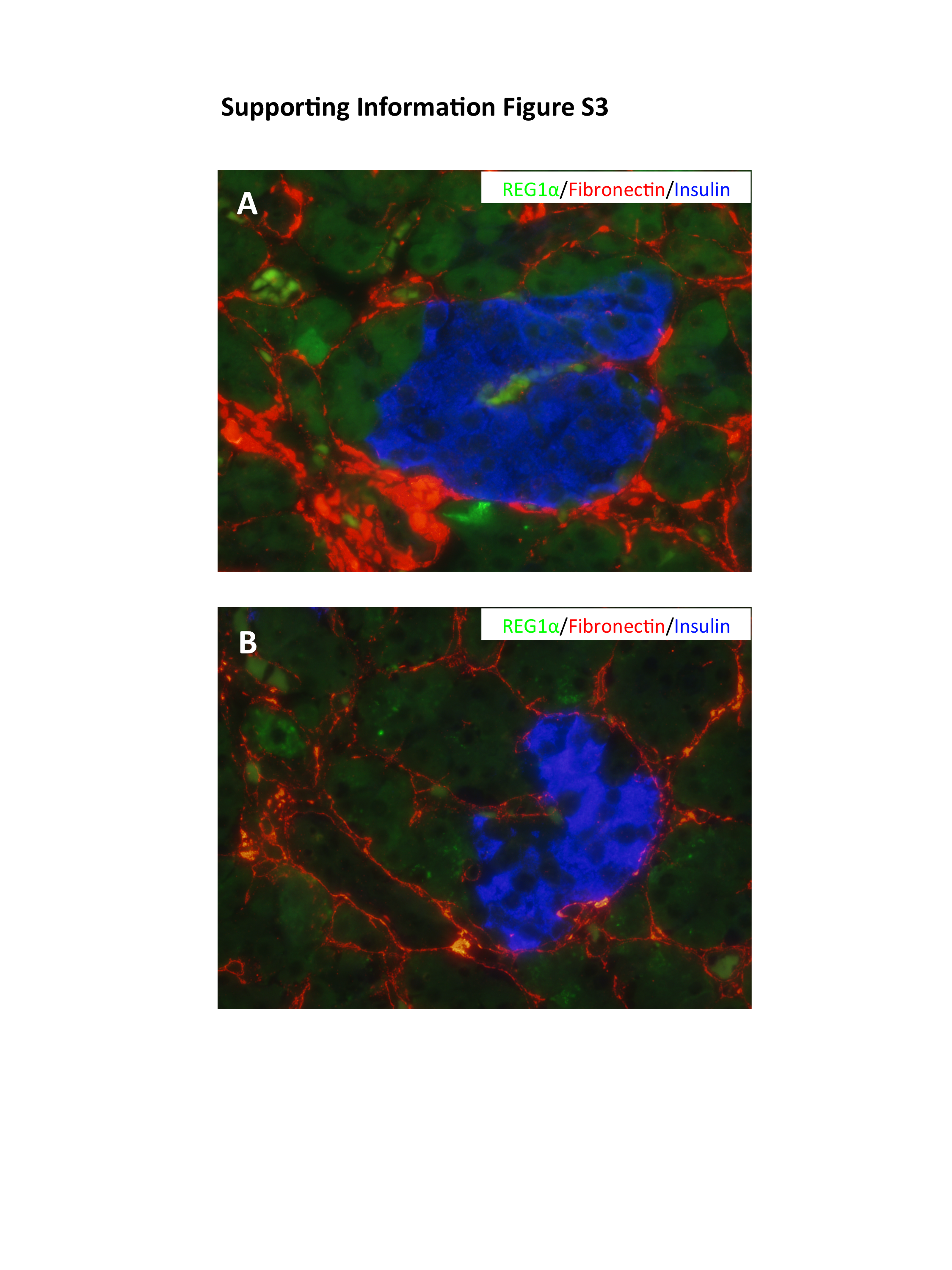

Supplement: Figure S3 — Acinar-like cell clusters touching Langerhans islets with thin interstitial surrounding (ATLANTIS) were found in pancreata of chronic pancreatitis (A) and type 2 diabetes (B). Over-expression of REG Iα was observed in chronic pancreatitis in both ATLANTIS cells and pancreatic acinar cells (A) but not in both ATLANTIS cells and pancreatic acinar cells of type 2 diabetes (B). (TIFF) [file pone.0095110.s003.tif]

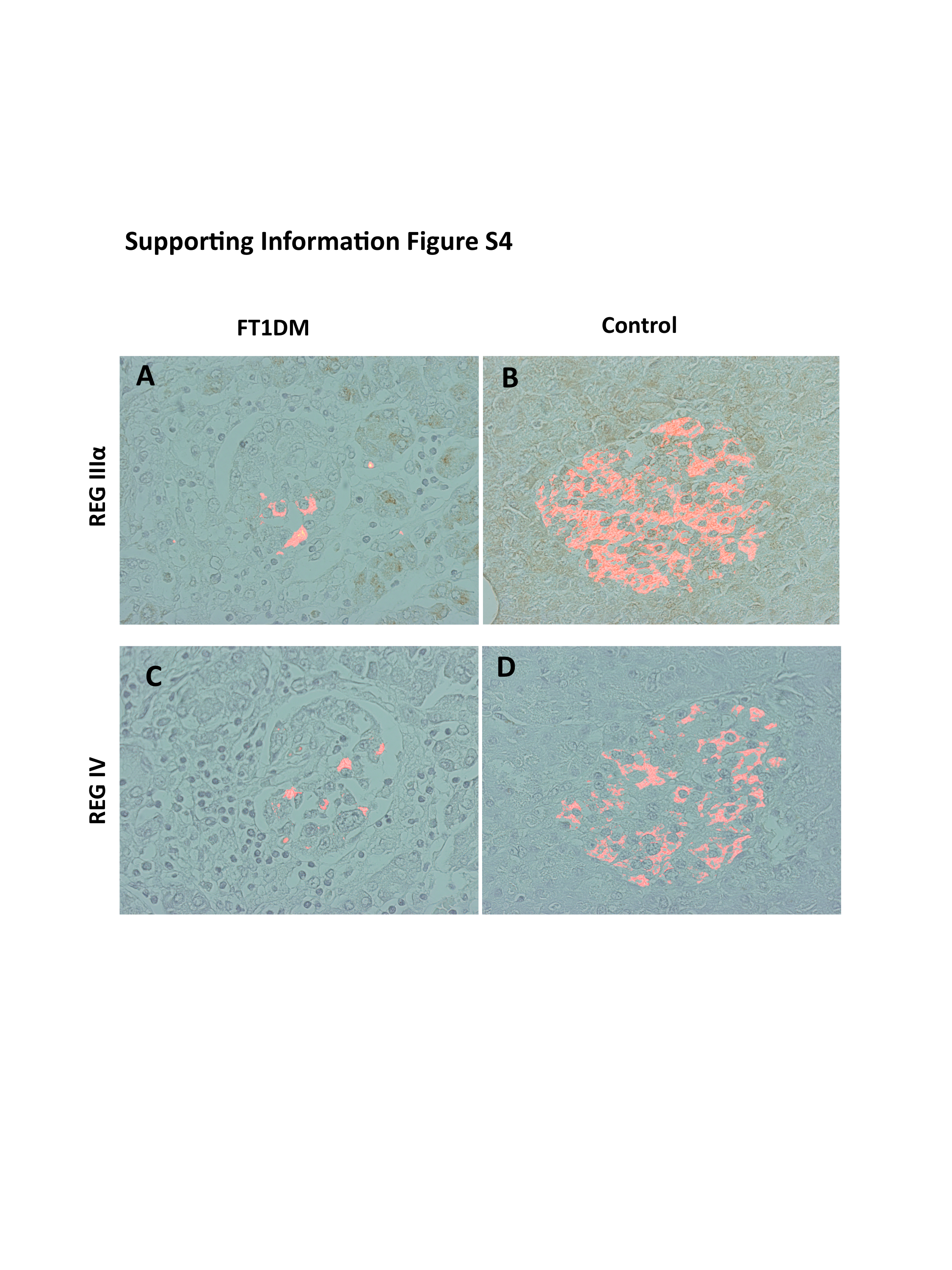

Supplement: Figure S4 — No over-expression of REG IIIα and REG IV was observed in the pancreas of FT1DM. Merged image of REG III (brown) and insulin (red) in FT1DM (A) and non-diabetic control (B). Merged image of REG IV (brown) and insulin (red) in FT1DM (C) and non-diabetic control (D). (TIFF) [file pone.0095110.s004.tif]

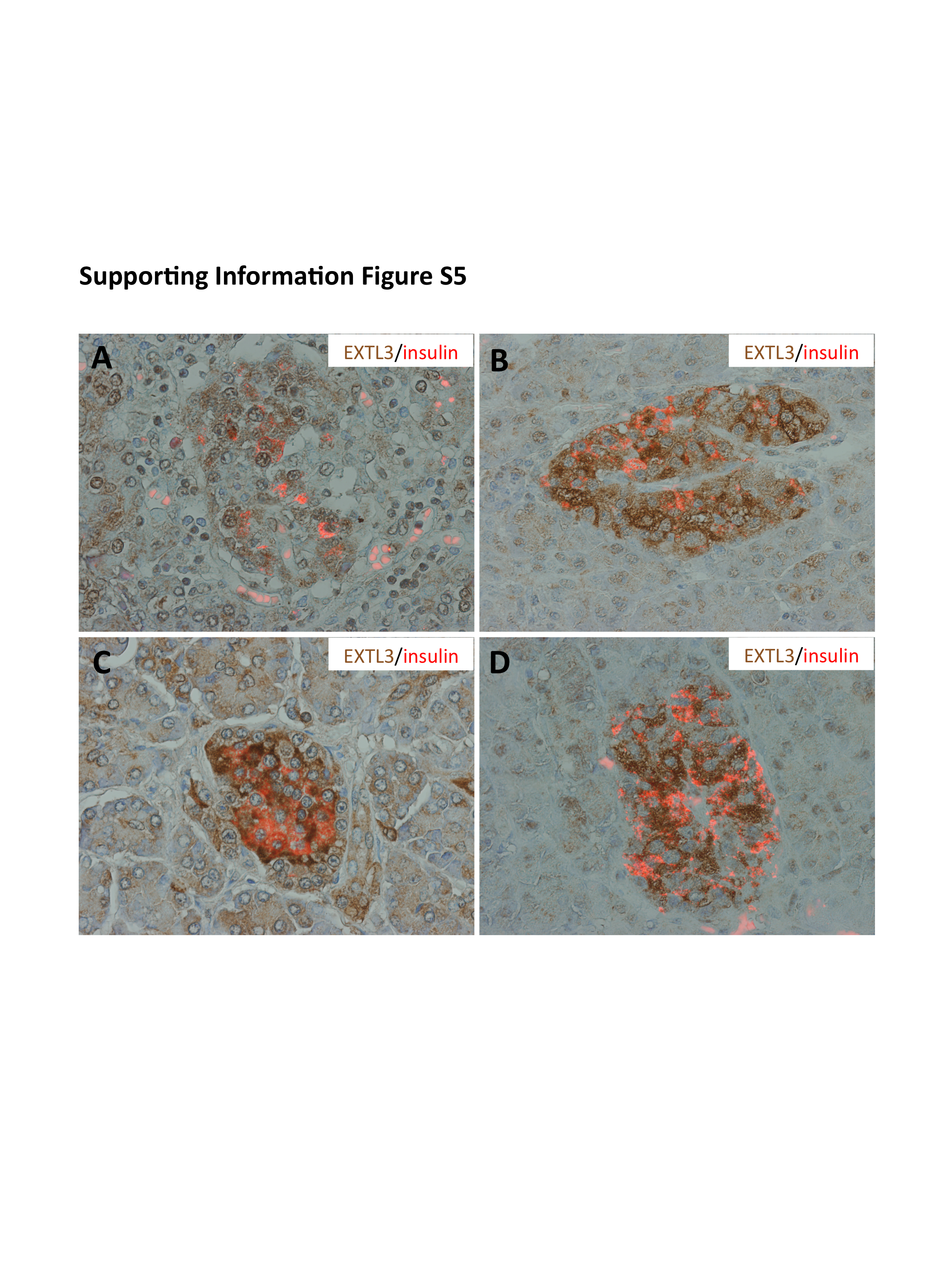

Supplement: Figure S5 — Expression of EXTL3 (brown), putative REG Iα receptor, was observed in beta cells (red) of fulminant type 1 diabetes (A), chronic pancreatitis (B), type 2 diabetes (C) and non-diabetic control (D). (TIFF) [file pone.0095110.s005.tif]
