# Supplementary material for: Distinct Cell Clusters Touching Islet Cells Induce Islet Cell Replication in Association with Over-Expression of Regenerating Gene (REG) Protein in Fulminant Type 1 Diabetes
Source: PLoS One. 2014 Apr 23;9(4):e95110. doi: 10.1371/journal.pone.0095110 (PMC3997392; doi:10.1371/journal.pone.0095110)
Supplement: Table S1 — Antibodies used in this study. (DOCX) [file pone.0095110.s006.docx]

**Supporting information Table S1.**

| **Antigen** | **Species** | **Clone** | **Source** |
| --- | --- | --- | --- |
| Insulin | Guinea pig | - | Dako, Carpinteria, CA |
| Insulin | Mouse | K36aC10 | Sigma, St. Louis, MO |
| Glucagon | Rabbit | - | Dako, Carpinteria, CA |
| Glucagon | Guinea pig |  | Linko, St. Charles, MD |
| Glucagon | Mouse | K79bB10 | Abcam, Cambridge, UK |
| Somatostatin | Mouse | SOM-018 | GeneTex, Irvine, CA, |
| Somatostatin | Rabbit |  | Dako, Carpinteria, CA |
| Pancreatic polypeptide | Goat |  | IMGENEX, SanDiego, CA |
| Pancreatic polypeptide | Rabbit |  | Dako, Carpinteria, CA |
| CD8 | Mouse | C8/144B | Dako, Carpinteria, CA |
| CD68 | Mouse | PG-M1 | Dako, Carpinteria, CA |
| REG Iα | Mouse |  | Gift, Dr. Shin Takasawa, Nara Medical University |
| REG Iα | Rabbit |  | Abcam, Cambridge, UK |
| Amylase | Goat |  | Santa Cruz Biotechnology, Santa Cruz, CA |
| Amylase | Mouse | G-10 | Santa Cruz Biotechnology, Santa Cruz, CA |
| Fibronectin | Rabbit |  | Sigma, St. Louis, MO |
| Collagen type IV | Mouse | COL-94 | Sigma, St. Louis, MO |
| Laminin | Rabbit |  | Sigma, St. Louis, MO |
| Ki67 | Rabbit |  | Abcam, Cambridge, UK |
| Cytokeratin 19 | Mouse | b170 | Novocastra, Newcastle Upon Tyne, UK |
| REG IIIα | Mouse | 512124 | R&D Systems, Minneapolis, MN |
| REG IV | Goat |  | R&D Systems, Minneapolis, MN |

EXTL3 Rabbit Gene Tex, Irvine, CA
